# Gene and domain shuffling in lytic cassettes of *Enterococcus* spp. bacteriophages

**DOI:** 10.1007/s13205-023-03775-w

**Published:** 2023-11-07

**Authors:** Jakub Viglasky, Maria Piknova, Peter Pristas

**Affiliations:** 1 Institute of Biology and Ecology, Faculty of Science, Pavol Jozef Šafárik University in Košice, Srobarova 2, 041 54 Košice, Slovakia; 2 Institute of Animal Physiology, Centre of Biosciences, Slovak Academy of Sciences, Soltesovej 4-6, 040 01 Kosice, Slovakia; 3 Laboratory of Biomedical Microbiology and Immunology, University of Veterinary Medicine and Pharmacy in Košice, 041 81 Košice, Slovakia

**Keywords:** Bacteriophage, Lytic cassettes, Gene shuffling, Domain shuffling, *Enterococcus*

## Abstract

The genomes of *Enterococcus faecalis* and *Enterococcus faecium* bacteriophages were analysed for gene shuffling in the lytic cassettes of bacteriophages infecting. It was found that enterococcal bacteriophages could be classified into well-defined groups based on the size of their genomes and each size group had its own conserved gene composition of lytic cassettes. Enterococcal bacteriophages use a relatively broad spectrum of holins and endolysins with variable cell-wall binding (CWB) and catalytic domains, and most of them utilise a lytic cassette with more than two genes. Enterococcal bacteriophages most commonly use endolysins with amidase catalytic domains and the CWB domain SH3_5. Some bacteriophages possess in their lytic cassette a holin-like gene with the XhlA domain protein, characteristic of hemolysin. Regardless of the shuffling of genes encoding holins and endolysins in lytic modules, a novel example of CWB domain shuffling within enterococcal endolysins was identified.

**Supplementary Information:**

The online version contains supplementary material available at 10.1007/s13205-023-03775-w.

## Introduction

Enterococci are part of the intestinal microflora of humans and domestic animals. Due to their exceptional adaptability, they can be isolated from various sources like soil, water, wild animals, and insects. The two most prevalent species, *Enterococcus faecalis* and *Enterococcus faecium*, are known to cause secondary infections in hospital environments. With the emergence of multidrug-resistant strains over the past two decades, they have become an important nosocomial pathogen causing urinary tract infections, bacteremia, and endocarditis (Fiore et al. 2019). Among enterococci, the major cause of infections is *E. faecalis*, but *E. faecium* is intrinsically more resistant to antibiotics. Enterococci show low-level resistance to cephalosporins and aminoglycosides and resistance to lincosamides and streptogramins. This intrinsic resistance gives them the ability to survive for longer periods of time in clinical environments and therefore acquire additional resistance from mobile genetic elements. The extensive use of antibiotics has caused the selection for antibiotic-resistant enterococci both in humans and in domestic animals (Hammerum, 2012). In clinical enterococcal isolates, multiple mobile genetic elements, mainly plasmids and transposons, were identified (Hollenbeck, 2012), which are responsible for the rapid dissemination of antibiotic resistance among enterococci. Antibiotics, especially vancomycin-resistant *Enterococcus* (VRE) species, have become one of the major nosocomial pathogens worldwide (O’Driscoll and Crank, 2015), and there is an urgent need to develop new strategies for the treatment of enterococcal infections.

Bacteriophages are estimated to be the most widely distributed life forms in the biosphere, with around 10 million particles for every 1 cm3 of any ecological niche where bacteria can be found (Hendrix 2003). Because of their ability to kill bacterial cells and their host specificity, phages are one of the most promising alternatives for fighting antimicrobial resistance in medicine and agriculture (Aminov R. et al., 2017). Besides the bacteriophage itself, phage-encoded proteins can be used as antimicrobials. The lysis of the cell wall is essential for new phage particles to leave the bacterial cell. Lytic modules of bacteriophages usually consist of a pair of genes encoding for holin and endolysin. Endolysins are bacterial cell wall hydrolases that play an important role in the release of mature bacteriophages in the late stage of the lytic life cycle of phage infection. Holins produce pores in the bacterial inner membrane, enabling endolysins to reach peptidoglycan in the cell wall, and are important in the correct timing of lysis at the end of the phage lytic cycle (Wang et al. 2000; Young 2014). The use of endolysins is considered safe. As phage-derived proteins, endolysins cannot create issues with possible gene transduction similar to phages, and there are no concerns regarding the emergence of endolysin-resistant bacteria (Chang 2020).

Endolysins from bacteriophages infecting gram-positive bacteria have evolved to utilise modular design. A typical gram-positive endolysin consists of two functional domains: the C—terminal cell-wall binding (CWB) domain and the N—terminal catalytic domain. The CWB domain keeps endolysin tightly bound to the bacterial cell wall, while the catalytic domain cleaves specific bonds in peptidoglycan (Donovan 2012). The CWB domain was shown to stay bound to the cell wall even after cell lysis to prevent the destruction of neighbouring cells that have not yet been infected. The outer membrane of gram-negative bacteria prevents this kind of damage by limiting access to the cell wall from the outside, which might explain small single-domain endolysins from phages infecting gram-negative bacteria (Schmelcher et al. 2012). Due to their lytic activity, endolysins are considered promising novel therapeutics for the treatment of bacterial infections (Schmelcher et al., 2021). Artificial domain shuffling in phage-encoded endolysins can lead to novel combinations of CWB and catalytic domains with higher specificity and effectivity against antibiotic-resistant strains of bacteria. For example, recombinant endolysins derived from bacteriophages infecting *Listeria*, *Bacillus*, *Streptococcus*, and *Staphylococcus* were constructed (Schmelcher et al. 2011; Loeffler et al. 2003; Donovan 2012; Son et al. 2021). *Enterococcus* phages have been isolated from various sources, including human microflora, suggesting that they are well tolerated when used in phage therapy. Most phages infecting *E. faecalis* can also infect *E. faecium*, and vice versa. Most known isolates belong to dsDNA-tailed bacteriophages of the order *Caudovirales* (Duerkop et al. 2014). In vitro, endolysin PlyV12 from Φ1 bacteriophage showed high lytic activity against antibiotic-resistant *E. faecium* and *E. faecalis* (Yoong et al. 2004). Several other endolysins from enterococcal bacteriophages have already been tested for anti-enterococcal activity either directly (Swift et al. 2016) or after in vitro swapping of CWB and catalytic domains between different parent bacteriophages (Binte Muhammad Jai et al. 2020). The aim of the present work was to analyse the genetic organisation of lytic cassettes of enterococcal bacteriophages and to identify the extent of in vivo gene and module shuffling.

## Materials and methods

### Bacteriophage genome dataset creation

Complete genome sequences of *Enterococcus* spp. bacteriophages were obtained from the GenBank database (Benson et al., 2013) using the keywords "enterococcus phage". For the list of bacteriophage genomes used in this study, see Table 1. Bacteriophage sequences were sorted by their size in bp with Biopython (Cock et al. 2009). Most of the genes encoding holins and endolysins were identified using Biopython and a keyword search in GenBank records. The keywords used were "N-acetylmuramoyl-L-alanine amidase", "endolysin", "phage lysin," and "lysin" for endolysin genes, and "holin" for holin genes. Holin and endolysin genes listed as hypothetical proteins, as well as other genes located near lytic cassettes, were identified manually and confirmed using protein Blast (Altschul et al. 1990) and InterProScan5 (Jones et al. 2014). For the list of bacteriophage sequences used in this study, see Supplementary Information.

**Table 1:** Combinations of catalytic and CWB domains observed in lytic cassettes of enterococcal bacteriophages

Catalytic domain	CWB domain	N° of cassettes	% of cassettes
Amidase_2	Unknown	56	32.75
	SH3_5	28	16.37
	ZoocinA	22	12.87
	SH3_5 + SH3_3	1	0.58
Amidase_5	SH3_5	19	11.11
CHAP	Unknown	29	16.96
GH_25	LysM	14	8.19
	Unknown	2	1.17
**Total**		**171**	**100.00**

**Table 2:** Combinations of holin-endolysin pairs in lytic cassettes of enterococcal bacteriophages

Holin	Endolysin		N° of cassettes	% of cassettes
	Catalytic domain	CWB domain		
PH_1	Amidase_2	SH3_5	23	13.45
		ZoocinA	22	12.87
		Unknown	10	5.85
	GH_25	LysM	2	1.17
PH_Dp1	GH_25	LysM	11	6.43
PH_4_1	Amidase_2	Unknown	18	10.53
	GH_25	Unknown	2	1.17
		LysM	1	0.58
PH_5_2	CHAP	Unknown	29	16.96
	Amidase_2	SH3_3 + SH3_5	1	0.58
		SH3_5	5	2.92
		Unknown	1	0.58
Holin_SPP1	Amidase_2	Unknown	21	12.28
	Amidase_5	SH3_5	19	11.11
HolinBhlA	Amidase_2	Unknown	4	2.34
PH_2_2	Amidase_2	Unknown	2	1.17
**Total**			**171**	**100.00**

### Protein domain identification

For protein domain identification, InterProScan5 and the Pfam databases (El-Gebali et al. 2019) were used. Domain shuffling was identified with multiple sequence alignment using the MUSCLE algorithm (Edgar 2004) in MEGA X (Kumar et al. 2018). In the case of unknown domains, RaptorX (Källberg et al. 2012) secondary structure prediction server was used for the identification of putative domains and interdomain linkers in endolysins.

## Results

**Fig. 1:**
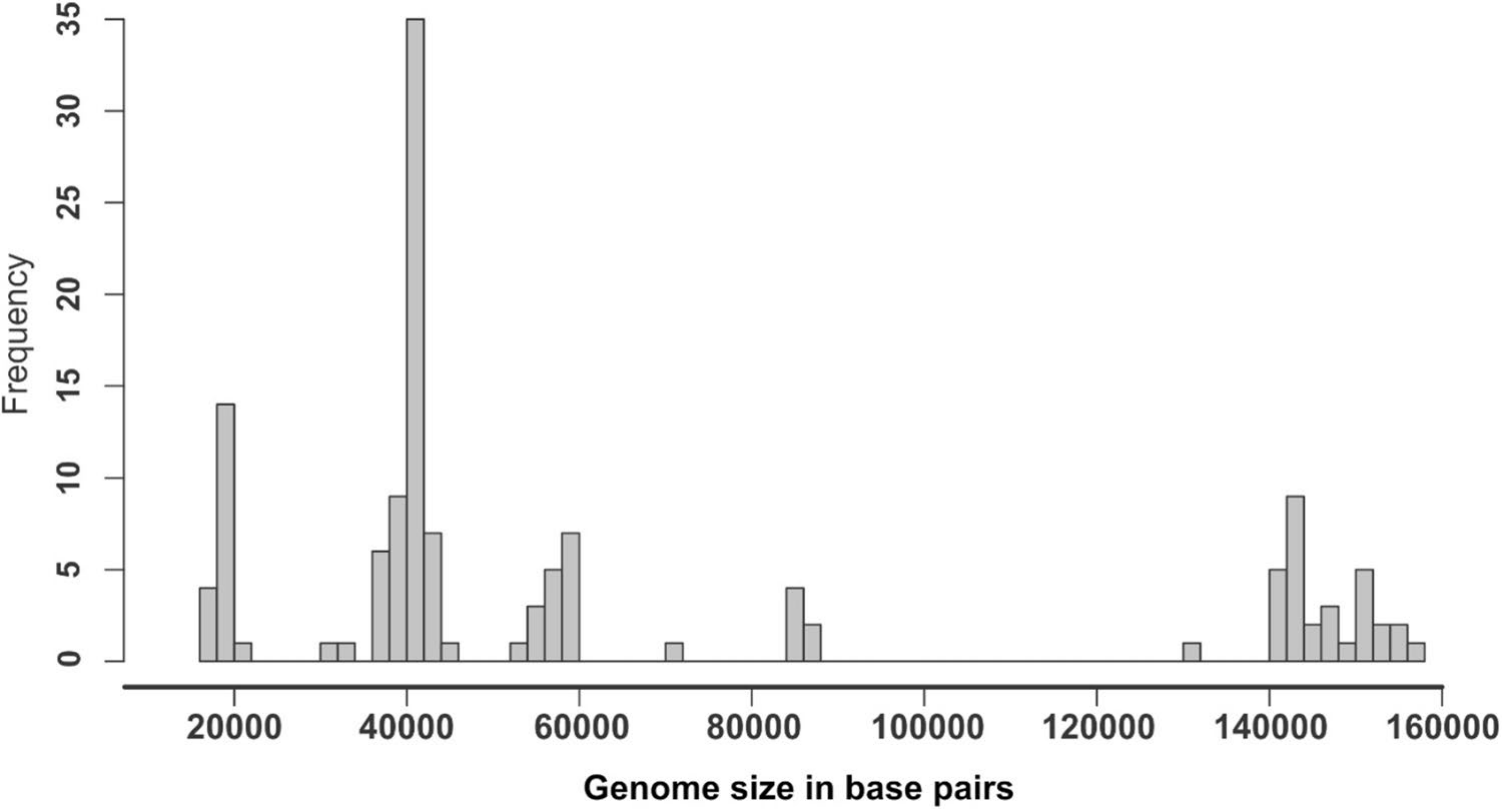
Histogram of Enterococcus spp. bacteriophage genome size distribution. Genomes of bacteriophages infecting Enterococcus spp. retrieved from the GenBank database were sorted by their size in bp. The size distribution was used to divide genomes into five size groups: Group 1, phages with genomes smaller than 20 kbp; Group 2, phages with genomes from 20 to 50 kbp; Group 3, phages with genomes from 50 to 65 kbp; Group 4, phages with genomes from 65 to 100 kbp; and Group 5, phages with genomes larger than 100 kbp

In our experiments, 171 complete genome sequences of dsDNA enterococcal bacteriophages were downloaded from the GenBank database and analyzed. Surprisingly, a clearly multimodal distribution of genome sizes of enterococcal bacteriophages was observed. The genomes could be sorted by their size into five groups: Group 1, phages with genomes smaller than 20 kbp; Group 2, phages with genomes from 20 to 50 kbp; Group 3, phages with genomes from 50 to 65 kbp; Group 4, phages with genomes from 65 to 100 kbp; and Group 5, phages with genomes larger than 100 kbp. The most frequent genome sizes in the range from 20 to 50 kbp were observed, accounting for nearly 50% of enterococcal bacteriophages (Fig. 1).

Genome annotations from GenBank records combined with multiple sequence alignments were used for the identification and gene composition of lytic cassettes. In lytic cassettes of enterococcal bacteriophages, 8 different holins were identified and their domains were found in the Pfam database: Phage_holin_4_1 (pfam: PF05105), Holin_BhlA (pfam: PF10960), Phage_holin_1 (pfam: PF04531), Phage_holin_Dp1 (pfam: PF16938), Phage_holin_2_2 (pfam: PF10746), Phage_holin_5_2 (pfam: PF16079), XhlA (pfam: PF10779), and Holin_SPP1 (pfam: PF04688).

Endolysins of enterococcal bacteriophages were found to possess 7 different catalytic domains already known to protein databases: Amidase_2 (pfam: PF01510), endopeptidase domain like (CATH Superfamily 3.90.1720.10), CHAP (pfam: PF05257), Glyco_Hydro_25 (pfam: PF01183), Amidase_5 (pfam: PF05382), Peptidase_M23 (pfam: PF01551), and Glucosaminidase (pfam: PF01832); and 4 types of CWB domains: ZoocinA_TRD (pfam: PF16775), SH3_3 (pfam: PF08239), SH3_5 (pfam: PF08460), and LysM (pfam: PF01476).

### Variability of lytic cassettes of enterococcal bacteriophages

**Fig. 2:**
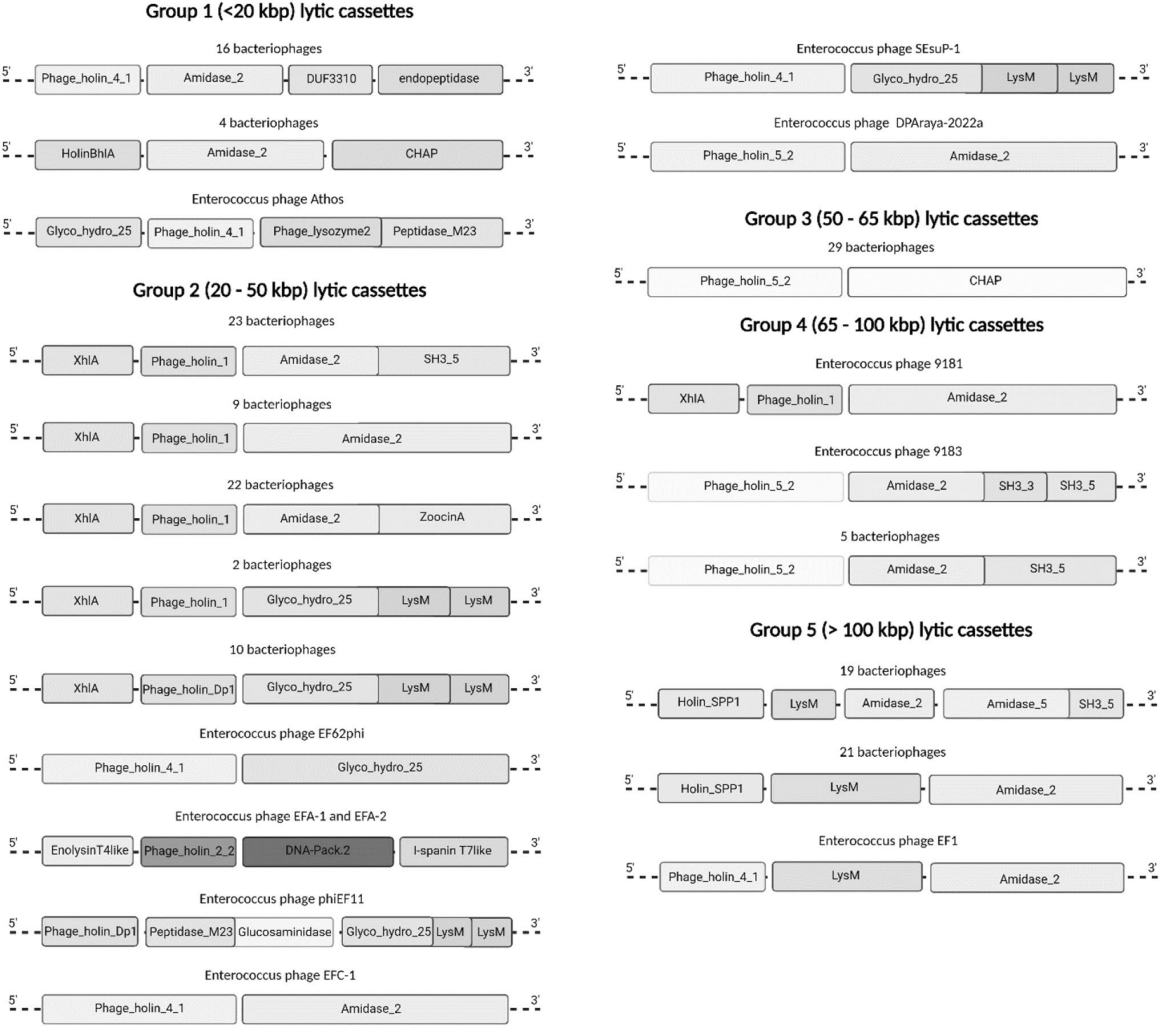
Variability of lytic cassettes in Enterococcus spp. bacteriophages. The most common lytic cassette composition in Group 1 was found in 15 bacteriophages, and only one phage in Group 1 had a unique lytic cassette. The most variable lytic cassette compositions were identified in Group 2 bacteriophages. Unique lytic cassettes were found in six bacteriophages. Group 3 was the most homogeneous and contained only one type of lytic cassette. Bacteriophages in Group 4 had lytic cassettes similar to those found in phages in Group 2. One bacteriophage had an endolysin with two CWB domains. Some of the Group 5 bacteriophages had lytic cassettes with two endolysins. Combinations of holins and endolysins with different catalytic and CWB domains were specific for each group

Based on sequence comparisons, there were 21 different combinations of proteins and their domains in lytic cassettes observed in enterococcal bacteriophages, and the distribution and composition of individual types of lytic cassettes were non-random regarding the size groups of bacteriophages. All types of lytic cassettes, together with corresponding numbers of bacteriophages possessing particular cassettes, are shown in Fig. 2.

The bacteriophages with the smallest genomes (Group 1, consisting of 21 bacteriophages with genome sizes of less than 20 kbp) had three types of lytic cassettes. The first type had holin with a Phage_holin_4_1 domain, followed by endolysin with an Amidase_2 domain, a protein with an unknown function, DUF3310, and an endopeptidase domain-containing protein. Based on multiple sequence alignments, Amidase_2 endolysin from phage MDA1 (MW623430.1) was used as the type endolysin for this lytic cassette. Secondary structure prediction showed that the Amidase_2 domain is connected with an unknown CWB domain via a proline-rich interdomain linker. The second type of lytic cassette in Group 1 contained holin with the HolinBhlA domain, endolysin with the Amidase_2 domain, and a CHAP (cysteine, histidine-dependent amidohydrolases/peptidases) domain-containing protein, which functions mainly in peptidoglycan hydrolysis. The predicted structure of Amidase_2 endolysin from this type of lytic cassette showed a similar proline-rich interdomain linker connecting another unknown CWB domain. Only one bacteriophage had the third type of lytic cassette consisting of protein with domains Phage_lysozyme2 and Peptidase_M23, holin with domain Phage_holin_4_1, and a second lytic protein with the Glyco_hydro_25 catalytic domain.

Group 2, consisting of 73 bacteriophages with genome sizes ranging from 20 to 50 kbp, was the most diverse in lytic cassette types. Most of the lytic cassettes had an endolysin with an N-terminal Amidase_2 domain, followed by a C-terminal SH3_5 or ZoocinA CWB domain, and a holin with a Phage_holin_1 domain. In some of these lytic cassettes, no known CWB domain was identified using InterProScan, but multiple sequence alignment showed that multiple different Amidase_2 sequences contained various C-terminal sequences, probably representing unknown CWB domains. Bacteriophages MSF2 (MK982307.1) and AUEF3 (KJ127304.1) were used for secondary structure prediction of endolysins with SH3_5 and ZoocinA domains. Surprisingly, the predicted structure of endolysin from MSF2 revealed another possible CWB domain between Amidase_2 and SH3_5. The second most common type of lytic cassette comprises endolysin with N-terminal Glyco_hydro_25 catalytic domain, followed by two C-terminal LysM domains, and holin with Phage_holin_Dp1, except for two bacteriophages with Phage_holin_1. All lytic cassettes either with Amidase_2 or Glyco_hydro_25 domains had holin-like protein with XhlA hemolysin domain. Several bacteriophages had unique lytic cassettes (Fig. 2).

Group 3 comprised 29 bacteriophages with only one type of lytic cassette. Holin exhibited 33.29% sequence similarity with the Phage_holin_5_2 domain of Group 4 lytic cassettes, and endolysin had only one CHAP domain and an unknown CWB domain. Lytic cassettes of this type were found only in bacteriophages in this group.

Group 4 comprised seven bacteriophages with lytic cassettes similar to those of Group 1 bacteriophages. Bacteriophage 9181 (MT939240.1) was the only one with a holin-like protein with an XhlA domain, a holin with a Phage_holin_1 domain, an endolysin with an N-terminal Amidase_2 domain, and an unknown C-terminal CWB domain. Although this phage belongs to size Group 4, multiple sequence alignment of Amidase_2 endolysins clearly showed that its endolysin is almost identical to endolysins from phages Aramis (LR990833.1) and dArtagnan (LR991625.1) from Group 2. The rest of the bacteriophages had holin with the Phage_holin_5_2 domain and endolysin with N-terminal Amidase_2 domain and C-terminal SH3_5 CWB domain. Multiple sequence alignment showed that bacteriophage nattely (MT119360.1) had the same sequence of the Amidase_2 domain as other phages in this group but a slightly different sequence of the putative CWB domain. However, the predicted secondary structure showed a surprisingly similar domain composition with the Amidase_2 catalytic domain, a putative unknown CWB domain, and the SH3_5 CWB domain. Bacteriophage 9183 (MT939241.1) had a unique amino acid sequence of endolysin with an Amidase_2 domain and two CWB domains, SH3_5 and SH3_3. The secondary structure of this endolysin resembled that of MSF2 (Group 2), nattely phage, and other phages from Group 4, but their amino acid sequences were different.

Group 5 was formed by 41 bacteriophages encoding three types of lytic cassettes. All three types had endolysin with an N-terminal Amidase_2 domain, an aggregation-promoting factor containing a LysM domain, and holin with a Holin_SPP1 domain, except for one bacteriophage with a Phage_holin_4_1 domain similar to the holins of Group 1. All bacteriophages from this group had a similar composition of lytic cassettes, but sequence analysis of endolysins revealed that there were two different types of endolysins with the Amidase_2 domain. The first was a protein with a shorter sequence of 289 amino acid residues. The second type had the same N-terminal amidase domain but consisted of 416 amino acid residues. Multiple sequence alignment and secondary structure prediction showed that there were CWB domains not previously identified by protein databases. Surprisingly, bacteriophages with longer endolysin contained another protein in their lytic cassette with an Amidase_5 catalytic domain and an SH3_5 CWB domain.

In summary, the most common catalytic domain was Amidase_2, followed by the CHAP domain, the Amidase_5 domain, and the Glyco_hydro_25 domain. Considering only known domains from the Pfam database, the most common combination of catalytic and CWB domains was found to be Amidase_2 + SH3_5, followed by Amidase_2 + ZoocinA, Amidase_5 + SH3_5, and Glyco_hydro_25 + LysM (Table 1). Combinations of holins with different endolysins were specific for each group of bacteriophages. The most common holin was Phage_holin_1 in a lytic cassette with an Amidase_2 domain containing endolysins. All holin-endolysin pair combinations are summarised in Table 2. As shown in Fig. 2, the types of lytic cassettes are unique for each group of bacteriophages. Although some Group 2 and Group 4 bacteriophages share the same domain composition of endolysin, multiple sequence alignments of endolysins with the Amidase_2 domain showed that those endolysins did not have the same amino acid sequence. Some endolysins from Group 2 bacteriophages share the same Amidase_2 domains with differences in their CWB domains, suggesting possible domain shuffling.

### Hemolysin-like proteins in lytic cassettes of Group 2 bacteriophages

Many bacteriophages from Group 2 with either Amidase_2 domain endolysins or Glyco_hydro_25 domain endolysins had in their lytic cassettes the holins Phage_holin_1 and Phage_holin_Dp1. However, GenBank records for another holin-like gene were found in the majority of enterococcal bacteriophage genomes from Group 2 with those specific lytic cassettes. Analysis using InterproScan revealed that this holin-like protein contained an XhlA hemolysin domain. This gene was always present in combination with Phage_holin_1 and Phage_holin_Dp1. However, in many genome annotations, this gene was misidentified as "tail fiber protein" or simply denoted as "hypothetical protein". Close localization of these holin-like protein genes to genes encoding holins might suggest that this gene plays an important role in bacteriophage lytic activity as well.

Gene shuffling in lytic cassettes of Group 2 and Group 5 bacteriophages In most lytic cassettes, holins were always paired with specific endolysins. Holins were also highly specific for each group. Surprisingly, some holins as well as endolysins were found in groups in which they were not expected. Some genes encoding holins were shuffled among bacteriophages with similar but not identical compositions of lytic cassettes. Gene shuffling was identified in lytic cassettes of bacteriophages from Group 2 and Group 5.

**Fig. 3:**
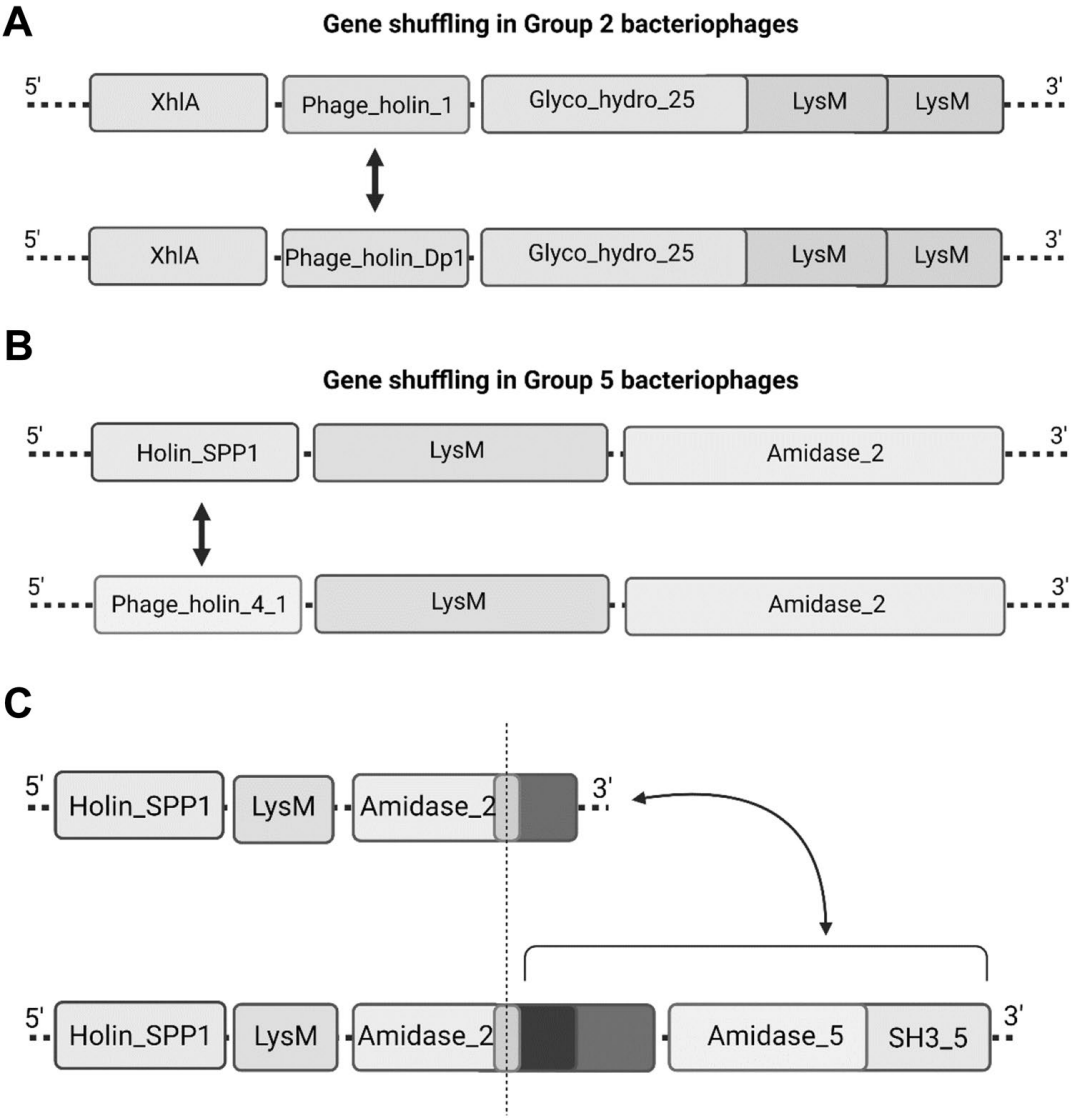
**A** Shuffling of holin-encoding genes in lytic cassettes of Group 2 bacteriophages; **B** Shuffling of holin-encoding genes in lytic cassettes of Group 5 bacteriophages; **C** Possible gene and domain shuffling in Group 5 bacteriophages. The CWB region of endolysin was probably inserted in lytic cassettes with a short variant of Amidase_2 containing endolysin along with another endolysin with Amidase_5 and SH3_5 domains

Two types of lytic cassettes were detected in Group 2 bacteriophages, consisting of identical endolysins with Glyco_hydro_25 catalytic domains and LysM CWB domains. These bacteriophages mostly encoded Phage_holin_Dp1, which we considered specific for this holin-endolysin pair. However, two of those bacteriophages were found to use Phage_holin_1 instead. Both lytic cassettes contained an XhlA holin-like protein. (Fig. 3A).

A similar situation could be seen in Group 5 bacteriophages, which contained endolysin with an Amidase_2 domain and a separate protein containing a LysM CWB domain. All bacteriophages in Group 5 used HolinSPP1 except one, which had Phage_holin_4_1 in its lytic cassette (Fig. 3B).

We also discovered that there were two types of Amidase_2 endolysins in Group 5. The endolysin with the longer amino acid sequence was present in lytic cassettes with another endolysin consisting of Amidase_5 and SH3_5 domains. Both types of lytic cassettes, the ones with longer Amidase_2 and Amidase_5 endolysins and the ones with shorter Amidase_2, had HolinSPP1. Multiple sequence alignments showed that the Amidase_2 domain regions of these endolysins were almost identical in sequence but differed in their putative CWB domain regions. This suggests that both gene and domain shuffling might have occurred in the evolution of the largest enterococcal bacteriophages (Fig. 3C).

### Domain shuffling

**Fig. 4:**
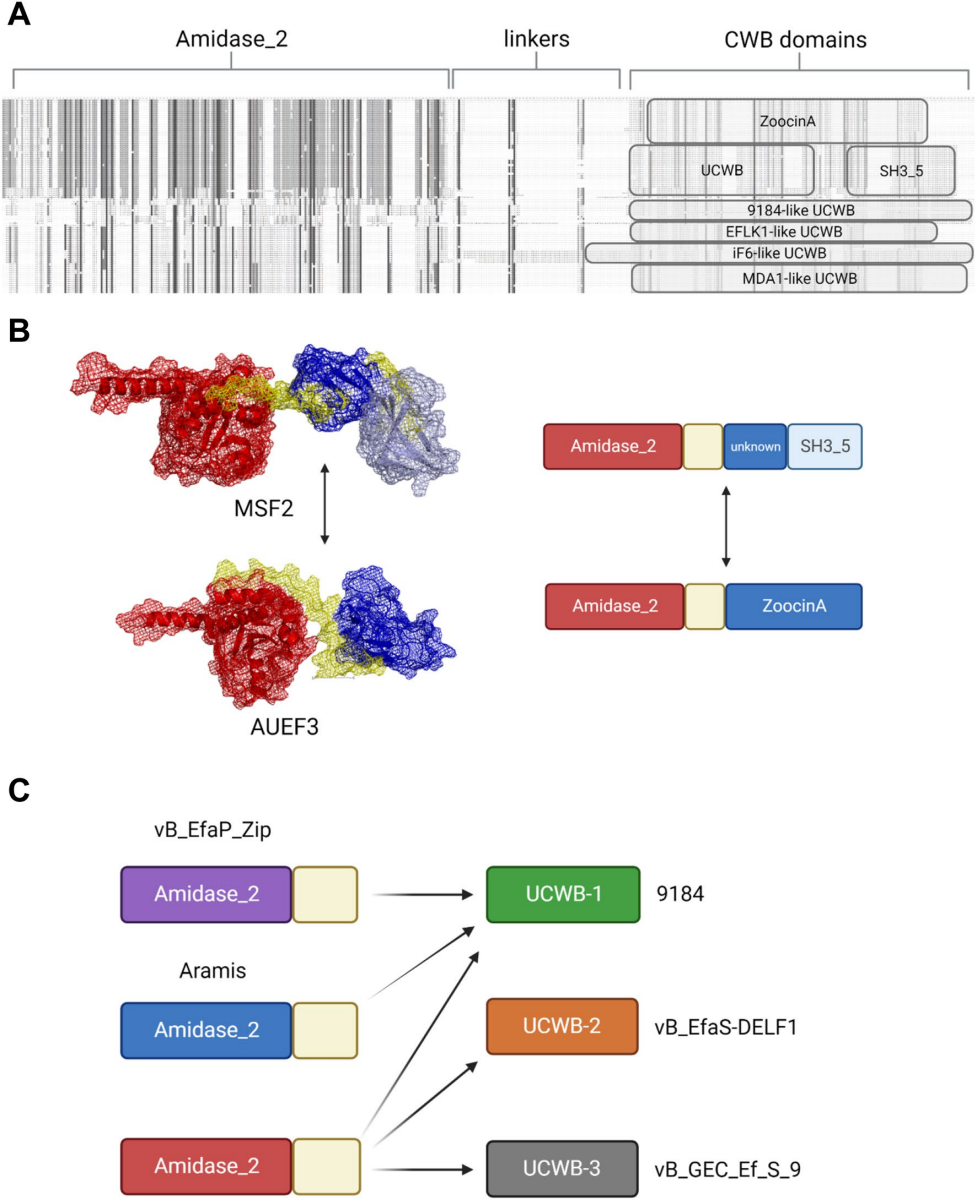
**A** Multiple sequence alignment of endolysins from all size groups containing Amiadase_2 catalytic domain. CWB domains ZoocinA, SH3_5, and putative unknown CWB domains are highlighted. The 9184-like UCWBs are UCWB-1, UCBW-2, and UCWB-3. **B** Domain shuffling in Group 2 bacteriophages. Endolysins from phage AUEF3 (KJ127304.1) and phage MSF2 (MK982307.1) represent all ZoocinA and SH3_5 domain containing endolysins from Group 2. Clearly, shuffling between domains ZoocinA and SH3_5 happened around the conserved linker sequence. **C** Possible domain shuffling in Group 1 and Group 2 bacteriophages. Bacteriophages from Group 2 had the same Amidase_2 domain (red) but different UCWB domains. Some bacteriophages with different Amidase_2 domains (purple for Group 1 and blue for Group 2) had the same UCWB1 domain. Bacteriophages from Group 1 had a UCWB1 domain like phage 9184 from Group 2

Since the most common catalytic domain used in endolysins of enterococcal bacteriophages is Amidase_2, and most of them contain unknown CWB domains, multiple sequence alignment of these endolysins was used to identify their differences. Based on multiple sequence alignment, 13 different endolysins with the Amidase_2 domain were identified, and only 4 of them had CWB domains that could be identified using InterProScan and the Pfam database. As expected, highly conserved amino acid residues were present in the Amidase_2 domain regions of endolysin sequences. Conserved residues found in CWB domains SH3_5 and ZoocinA were present in most sequences with unknown CWB domains, suggesting that even unidentifiable parts of the endolysins were most likely to be CWB domains. Although Amidase_2 domains and CWB domains were not identical in all endolysins, sequences of putative interdomain linkers were highly conserved in all endolysins containing Amidase_2 domains (Fig. 4A). The linker sequences following the Amidase_2 domain were rich in proline and lysine residues, with the consensus sequence N–KPTKPPSKPPPKP–C. Endolysins containing the SH3_5 CWB domain had an amino acid sequence longer than expected for the SH3_5 domain, suggesting the existence of another not yet identified domain between the putative linker and the SH3_5 domain. ZoocinA-containing endolysins supported this idea because the ZoocinA domain followed right after the linker sequence without an "empty" sequence in between. For that reason, secondary structure prediction was used to identify these putative unknown CWB domains.

Endolysins from Group 2 bacteriophages with CWB domains SH3_5 and ZoocinA had almost identical sequences of Amidase_2 domains. Endolysins from bacteriophages MSF2 and AUEF3 were used as representatives for SH3_5 and ZoocinA-containing endolysins and were used for secondary structure prediction. Predicted secondary structures confirmed the highly conserved flexible interdomain linker and showed that the SH3_5 domain containing endolysins had another small putative CWB domain. Since the Amidase_2 domain sequence in those endolysins was almost identical and the main difference was in CWB domains, domain shuffling between putative CWB domain with SH3_5 domain and ZoocinA domain most likely happened in related bacteriophages from Group 2 (Fig. 4B).

The rest of the Amidase_2 endolysins in Group 2 did not have known CWB domains, but based on multiple sequence alignment, three putative CBW domains were identified in endolysins with two different Amidase_2 domains. For further analysis of these domains, representative endolysins from bacteriophages 9184 (MT939242.1), vB_EfaS-DELF1 (LC513943.1), vB_GEC_Ef_S_9 (MW672041.1), and Aramis (LR990833.1) were used. Endolysins from bacteriophages 9184, vB_EfaS-DELF1, and vB_GEC_Ef_S_9 had the same amino acid sequence of the Amidase_2 domain with differences in their putative CWB domain regions. Putative CWB domains were denoted as UCWB (unknown CWB) domains. Secondary structure predictions of representative endolysins showed that those UCWB domain regions contained large CWB domains as opposed to SH3_5-containing endolysins. Since the Amidase_2 domain and interdomain linkers had high sequence similarity, domain shuffling may have happened between UCWB domains in these endolysins. Surprisingly, endolysin from bacteriophage Aramis had a different Amidase_2 domain but the same UCWB as endolysin from bacteriophage 9184. That suggests possible shuffling not only between CWB domains but between catalytic domains as well. The putative interdomain linker of this endolysin slightly differed from the consensus sequence. The same Amidase_2 and UCWB domains were found in Group 4 bacteriophage 9181. A similar situation probably happened with bacteriophages vB_EfaP_Zip (MK360025.1) and vB_EfaP_IME199 (KT945995.1) from Group 1. Their endolysin contained the same UCWB domain as previously mentioned bacteriophages 9184 and Aramis but a completely different Amidase_2 domain (Fig. 4C).

**Fig. 5:**
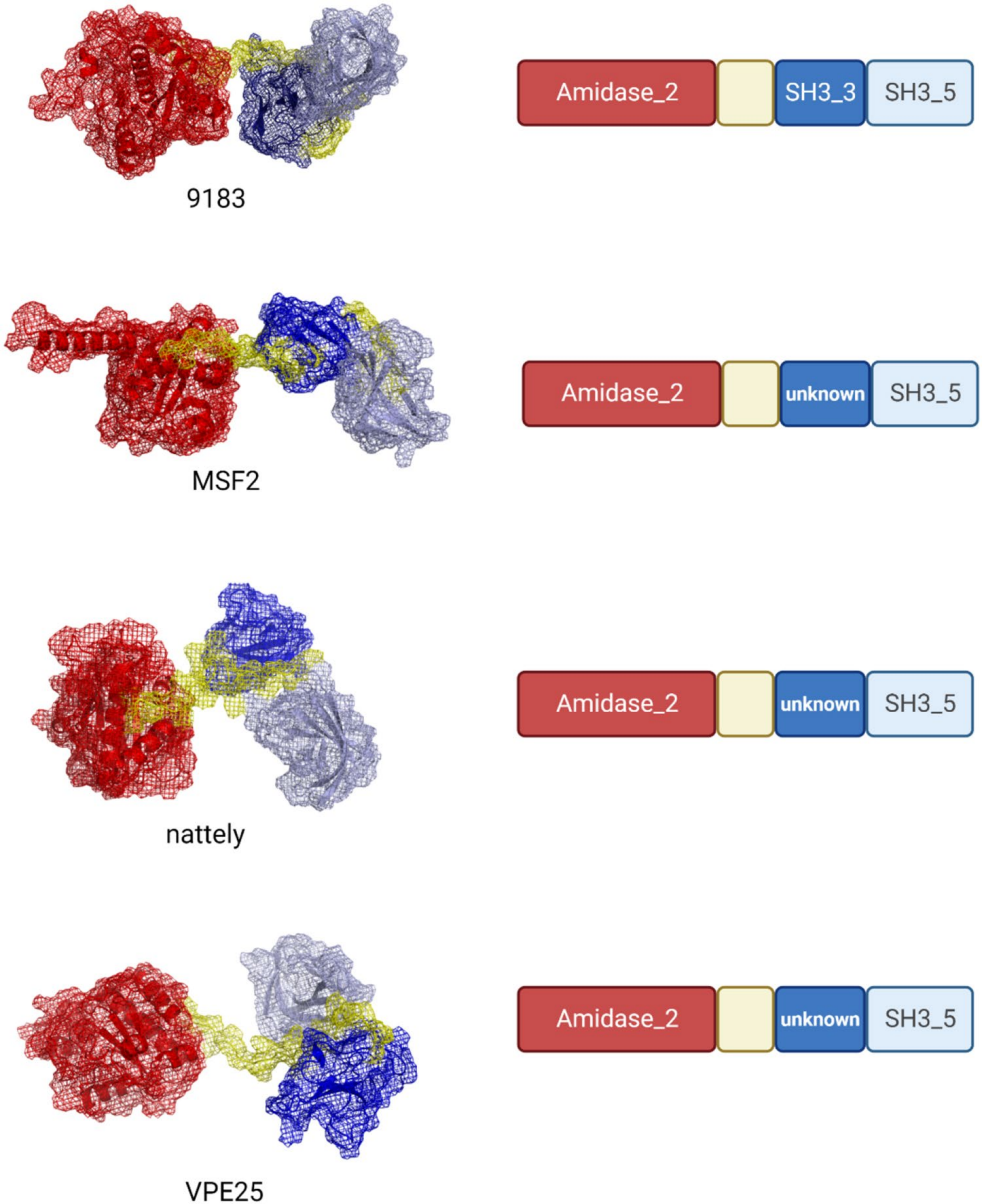
Predicted secondary structures of Group 2 and Group 4 bacteriophages. Endolysin from bacteriophage MSF2 represents all endolysins from Group 2 bacteriophages with Amidase_2 and SH3_5 domains. Despite having different amino acid sequences, these endolysins have similar predicted structures with possible unknown CWB domain. Only bacteriophage 9183 has two CWB domains known to protein databases

Endolysins from Group 4 bacteriophages with domain composition similar to that of Group 2 endolysins with the SH3_5 domain showed possible domain shuffling based on multiple sequence alignment. Secondary structure prediction of endolysins from bacteriophages nattely and VPE25 was performed because their sequence alignment suggested domain shuffling between the unique nattely endolysin and other endolysins from Group 4. The structure prediction of endolysin from phage 9183 was performed as this endolysin was the only one with two known CWB domains, SH3_3 and SH3_5. Predicted structures revealed that, despite sequence differences, the structure of endolysins in Group 4 was similar, with a C-terminal SH3_5 domain and a putative unknown CWB domain. Even endolysin from the 9813 bacteriophage with little sequence similarity to nattely and VPE25-like endolysins had a similar predicted secondary structure (Fig. 5).

Unknown CBW domains were also identified in Amidase_2 endolysins from Group 5 bacteriophages (Fig. 4A). Bacteriophages with Amidase_5 + SH3_5 endolysin in their lytic cassette had longer Amidase_2 endolysin than those without Amidase_5 containing endolysin. Multiple sequence alignment showed a similar Amidase_2 domain, and the difference in length was in the CWB region of the sequence. That suggested possible domain shuffling between unknown CWB domains. Since endolysins with SH3_5 domains from Group 2 and Group 4 had longer sequences because of previously unidentified CWB domains, secondary structure prediction was used for Group 5 endolysins to identify putative CWB domains in shorter and longer Amidase_2 endolysins. Endolysin from bacteriophage EFLK1 (KR049063.1) was used for secondary structure prediction of the shorter variant, and endolysin from bacteriophage iF6 (MT909815.1) for the longer variant. Predicted structures clearly showed two large CWB domains in iF6 Amidase_2 endolysin and only one small domain in EFLK1 Amidase_2 endolysin. The shuffling probably happened between sequences downstream of the conserved linker, with one small CWB domain being replaced by two CWB domains and additional endolysin (Fig. 3C).

## Discussion

In total, 171 bacteriophage genome sequences from *Enterococcus* spp. were analyzed. A comparison of their sizes in bps showed that the sequences can be divided into five separate groups. Such multimodal distribution of bacteriophage genome sizes has been reported e.g., for prophages of *Acinetobacter baumanii* (Loh et al. 2020). Although there is no clear explanation for this observation, it can be hypothesised that the genome size distribution reflects the geometry and packaging capacity of bacteriophage heads.

Lytic cassettes of all bacteriophages were identified in GenBank records using a keyword search. In addition to the classic canonic structure of a lytic cassette containing a holin-lysin pair (Young 2014), some of the studied lytic cassettes contain multiple proteins, probably involved in the lytic cycle. More than two proteins in the lytic cassette are typical for lambdoid phages, which use holin-independent lysis mechanisms (Briers et al. 2011).

Combinations of 7 catalytic and 4 CWB domains were identified in *Enterococcus* phages. The most common catalytic domain was Amidase_2 (62.57% of lytic cassettes). Most Amidase_2 domain-containing endolysins had unknown CWB domains (32.75% of lytic cassettes). Domain SH3_5 was found to be typical in the Amidase_2 endolysins of enterococcal bacteriophages. The common lytic cassettes of *Enterococcus* spp. phages were different from those of other *Lactobacillales* phages. The combination of Amidase_2 + SH3_5 domains is typical for *Enterococcus* phages and prophages. *Lactobacillus* prophages lytic cassettes have the majority of endolysins with domains Glyco_hydro_25 + LysM (Maliničová et al. 2012). This domain is also found in a minority of *Enterococcus* phages, as shown in our results.

Lytic cassettes of *Enterococcus* phages contained 7 types of holins, with the Phage_holin_1 domain being the most common. This type of holin formed lytic cassettes with the most common endolysin, so Phage_holin_1 + Amidase_2 + SH3_5 was the most typical lytic cassette in Enterococcus phages. Almost all lytic cassettes with Phage_holin_1 and Phage_holin_Dp1 contained another holin-like protein with the XhlA hemolysin domain. This domain was found in the hemolysin of the gamma-proteobacterium *Xenorhabdus nematophila* and is responsible for the lysis of insect granulocytes, plasmacytes, and rodent erythrocytes (Cowles et al. 2005). Holin-like protein with this domain was also found in the lytic cassette of *Bacillus subtilis* prophage PBSX. The whole PBSX lytic cassette contains genes for XhlA holin-like protein, XhlB holin, and XlyA endolysin. Expression of all three proteins is essential for the lysis of the host cell. XhlA probably forms a membrane complex with XhlB, which is necessary for endolysin release through the membrane (Longchamp et al. 1994; Krogh et al. 1998). A similar composition of the lytic cassette was found in *Enterococcus* phages from Group 2, including SHEF phages (Al-Zubidi et al. 2019), IME-EFm1 (Wang et al. 2014), and IME-Efm5 phages (Gong et al. 2016). Our findings show that in *Enterococcus* phages, XhlA is always associated with Phage_holin_1 or Phage_holin_Dp1 in lytic cassettes.

Combinations of holins and endolysins are specific for groups of phages. Phages from Group 2 with Glyco_hydro_25 domain endolysin prefer Phage_holin_Dp1. Two phages with this type of endolysin use Phage_holin_1, suggesting that gene shuffling has occurred between phages with a similar holin-endolysin pair and the XhlA domain. Different gene shuffling happened in phages from Group 5. All bacteriophages from Group 5 had a holin with the HolinSPP1 domain, except one with Phage_holin_4_1, similar to bacteriophages from Group 1. All bacteriophages from Group 5 contained Amidase_2 endolysin, a protein with LysM domain and HolinSPP1, but some had in their lytic cassette another endolysin with Amidase_5 and SH3_5 domains. However, multiple sequence alignment revealed that there were two types of Amidase_2 endolysins in Group 5. These two types differed in their CWB domains, and only lytic cassettes with longer Amidase_2 endolysin contained Amidase_5 + SH3 + 5 endolysin. Domain and gene shuffling were identified in those types of lytic cassettes. The Amidase_5 domain in endolysin was found to be typical for *Streptococcus* phages (Maliničová et al. 2012) and is widely used in vitro for the construction of endolysins with improved host spectrum and activity.

Domain shuffling was identified in endolysins with the Amidase_2 domain from all size groups of enterococcal bacteriophages. Multiple sequence alignment as well as secondary structure predictions were used to identify differences in Amidase_2 domains and putative CWB domains in endolysins where no known CWB domain was identified. This approach showed that apart from domain shuffling in endolysins with SH3_5 and ZoocinA domains, there probably were more shuffling events in the evolution of lytic cassettes of enterococcal bacteriophages. Putative UCWB domains were shuffled in bacteriophages from all size groups containing Amidase_2 endolysins. Three UCWB domains were identified in Group 2 bacteriophages. Some of the shuffling events probably happened between catalytic instead of CWB domains of endolysins, as they differed in the sequence of Amidase_2 domains but had identical UCWB domains. Gene and domain shuffling identified in Group 5 bacteriophages not only changed the CWB domains of Amidase_2 endolysins but also inserted another endolysin with an Amidase_5 domain. Based on predicted secondary structures and sequence alignments, putative interdomain linkers with highly conserved residues were identified as probable sites where domain shuffling happened. Similarly, shuffling was identified in different *Lactobacillales* prophages (Maliničová et al. 2012).

Our results provide useful information that can be used for the production of chimeric endolysins with antimicrobial activity. Swapping of catalytic and CWB domains has been done to produce chimeric endolysins with better activity against multidrug-resistant Staphylococcus aureus. However, only 12 naturally occurring endolysins were used for library construction (Son et al. 2021).

With the growing number of available bacteriophage genome sequences, we can have a better understanding of naturally occurring domain combinations in the lytic cassettes of bacteriophages infecting various species of bacteria. As shown in our work, the types of lytic cassettes and domains are probably species-specific, with some overlaps between related bacterial species. Available data and multiple sequence alignments should be used for a larger library of endolysins and various combinations of their domains. Cloning of this library and production of natural and chimeric endolysins in a suitable expression system would be the first step to confirm our in silico analysis results but also to confirm whether the unknown CBW domains are in fact CWB domains or have a different, not yet identified function. Different combinations active against *Enterococcus* spp. could be tested against related bacteria from the order *Lactobacillales* to select either highly specific endolysins or endolysins with broader specificity.

## Conclusion

Bacteriophages infecting *Enterococcus* spp. can be divided into five groups based on their genome size. Every group comprised bacteriophages with similar compositions of lytic cassettes. The most common endolysin for enterococcal phages contained the Amidase_2 catalytic domain and the SH3_5 CWB domain. Some lytic cassettes with Amidase_2 domain endolysin showed possible domain shuffling between CWB domains SH3_5 and ZoocinA and between unknown CWB domains. Our findings suggest that lytic cassette composition is specific for *Enterococcus* spp. phages, and different lytic cassettes might be used by phages infecting other species of bacteria. Identification of natural domain shuffling and putative interdomain linkers shows which domains should work in recombinant endolysins against *Enterococcus faecalis* and *Enterococcus faecium.* Identified catalytic and CWB domains and their natural and artificial combinations should be used in the future for the construction of chimeric endolysins to test which combination is the most active against *E. faecalis* and *E. faecium*. Putatively unknown CWB domains provide information about previously hidden diversity in phage endolysins. We believe that this diverse repertoire of possible highly specific domains is also present in bacteriophages infecting other bacteria and should not be overlooked in further research focused on finding new and effective antimicrobial therapeutics.

### Supplementary Information

Below is the link to the electronic supplementary material.

Supplementary file1 (DOCX 26 KB)

## Data Availability

All data used through this study were obtained from GenBank database and are freely available.
