# Supplementary material for: Gene and domain shuffling in lytic cassettes of Enterococcus spp. bacteriophages
Source: 3 Biotech. 2023 Nov 7;13(12):388. doi: 10.1007/s13205-023-03775-w (PMC10630273; doi:10.1007/s13205-023-03775-w)
Supplement: Supplementary file 1 — Supplementary file1 (DOCX 26 KB) [file 13205_2023_3775_MOESM1_ESM.docx]

| **Bacteriophage** | **GenBank**  **accession No.** | **Host** | **Genome size (bp)** |
| --- | --- | --- | --- |
| **Group 1** |  |  |  |
| AE4_17 | MT757399.1 | *E. faecalis* | 18477 |
| EFRM31 | GU815339.1 | *E. faecalis* | 16945 |
| Idefix | LT630001.1 | *Enterococcus* sp. | 18168 |
| vB_Efae230P-4 | JQ309827.1 | *E. faecium* | 17972 |
| vB_EfaP_Ef6.2 | MK721188.1 | *E. faecalis* | 17966 |
| vB_EfaP_Ef6.3 | MK721196.1 | *E. faecalis* | 18136 |
| vB_EfaP_Ef7.2 | MK721183.1 | *E. faecalis* | 18737 |
| vB_EfaP_Ef7.3 | MK721184.1 | *E. faecalis* | 18818 |
| vB_EfaP_Ef7.4 | MK721198.1 | *E. faecalis* | 18415 |
| vB_EfaP_Efmus1 | MK721195.1 | *E. faecalis* | 17927 |
| vB_EfaP_Efmus2 | MK721197.1 | *E. faecalis* | 18366 |
| vB_EfaP_Efmus3 | MK721185.1 | *E. faecalis* | 18286 |
| vB_EfaP_Efmus4 | MK721193.1 | *E. faecalis* | 18186 |
| vB_EfaP_IME195 | KT932700.2 | *E. faecalis* | 18655 |
| vB_EfaP_IME199 | KT945995.1 | *E. faecium* | 18838 |
| vB_EfaP_Zip | MK360025.1 | *E. faecium* | 18742 |
| ZEF1 | MT747434.1 | *E. faecalis* | 18454 |
| Athos | LR990834.1 | *Enterococcus* sp | 18345 |
|  |  |  |  |
| **Group 2** |  |  |  |
| AE4_2 | MT747435.1 | *E. faecalis* | 37561 |
| AUEF3 | KJ127304.1 | *E. faecalis* | 41257 |
| Ec-ZZ2 | KR131750.1 | *E. faecium* | 41170 |
| EF62phi | CP002495.1 | *E. faecalis* | 30505 |
| EFA-1 | MT350292.1 | *E. faecalis* | 40712 |
| EFA-2 | MT350293.1 | *E. faecalis* | 39964 |
| EfaCPT1 | JX193904.1 | *E. faecalis* | 40923 |
| EFC-1 | KJ608188.1 | *E. faecalis* | 40286 |
| heks | MT119359.1 | *E. faecalis* | 39708 |
| IME-EF4 | KF733017.1 | *Enterococcus* sp. | 40692 |
| IME-Efm1 | KJ010489.1 | *E. faecium* | 42597 |
| IME-Efm5 | KT588072.1 | *E. faecium* | 42265 |
| IME-EF3 | KF728385.2 | *E. faecalis* | 41687 |
| LY0322 | MH193369.1 | *E. faecalis* | 40934 |
| LY0323 | MH375074.1 | *E. faecalis* | 40876 |
| MSF2 | MK982307.1 | *E. faecalis* | 40880 |
| Nonaheksakonda | MK125140.1 | *E. faecalis* | 41994 |
| phiEf11 | GQ452243.1 | *E. faecalis* | 42822 |
| phiFL1A | GQ478081.1 | *E. faecalis* | 38764 |
| phiFL1B | GQ478082.1 | *E. faecalis* | 38989 |
| phiFL1C | GQ478083.1 | *E. faecalis* | 38721 |
| phiFL2A | GQ478084.1 | *E. faecalis* | 36270 |
| phiFL2B | GQ478085.1 | *E. faecalis* | 36826 |
| phiFL3A | GQ478086.1 | *E. faecalis* | 39576 |
| phiFL3B | GQ478087.1 | *E. faecalis* | 40275 |
| phiFL4A | GQ478088.1 | *E. faecalis* | 37856 |
| phiNASRA1 | MG264739.2 | *E. faecalis* | 40139 |
| phiSHEF2 | MF678788.1 | *E. faecalis* | 41712 |
| phiSHEF4 | MF678789.1 | *E. faecalis* | 41081 |
| phiSHEF5 | MF678790.1 | *E. faecalis* | 41598 |
| PMBT2 | MG708276.1 | *E. faecalis* | 41489 |
| Q69 | CAJDJX010000002.1 | *Enterococcus* sp. | 42141 |
| SANTOR1 | KX284704.1 | *E. faecalis* | 37933 |
| vB_EfaS-271 | MT520979.1 | *E. faecalis* | 40197 |
| vB_EfaS-DELF1 | LC513943.1 | *E. faecalis* | 40248 |
| vB_EfaS-159 | CAJDKF010000002.1 | *Enterococcus* sp. | 41718 |
| vB_EfaS_AL2 | MH203384.1 | *E. faecalis* | 40836 |
| vB_EfaS_AL3 | MH203383.1 | *E. faecalis* | 40789 |
| vB_EfaS_Ef5.1 | MK721199.1 | *E. faecalis* | 41141 |
| vB_EfaS_Ef5.2 | MK721186.1 | *E. faecalis* | 41418 |
| vB_EfaS_Ef5.3 | MK721200.1 | *E. faecalis* | 39115 |
| vB_EfaS_Ef5.4 | MK721191.1 | *E. faecalis* | 40685 |
| vB_EfaS_Ef6.1 | MK721187.1 | *E. faecalis* | 40429 |
| vB_EfaS-Ef6.4 | MK721190.1 | *E. faecalis* | 41133 |
| vB_EfaS_IME196 | KT932701.1 | *E. faecalis* | 38886 |
| vB_EfaS_IME197 | KT945994.2 | *E. faecalis* | 41098 |
| vB_EfaS_LM99 | MH355583.1 | *E. faecalis* | 40203 |
| vB_EfaS_Max | CAJDKB010000002.1 | *Enterococcus* sp. | 40542 |
| vB_EhiS_268 | MK360024.1 | *E. faecalis* | 40975 |
| EFP1 | MN995824.1 | *E. faecalis* | 37561 |
| EFAP-1 | FJ792813.1 | *E. faecalis* | 21115 |
| vB_EFaS_TV217 | MT627482.1 | *E. faecalis* | 41486 |
| vB_EFaS_TV51 | MT661597.1 | *E. faecalis* | 41821 |
| vB_EFaS_TV54 | MT661598.1 | *E. faecalis* | 42438 |
| FX417 | MT829326.1 | *E. faecalis* | 41061 |
| 9184 | MT939242.1 | *E. faecium* | 44108 |
| vB_EfaS-SRH2 | LC623721.1 | *E. faecalis* | 38749 |
| vb_GEC_Ef_S_9 | MW672041.1 | *E. faecium* | 33631 |
| vB_EfaS_785CC | MZ182245.1 | *E. faecalis* | 40956 |
| Aramis | LR990833.1 | *Enterococcus* sp | 42697 |
| dArtagnan | LR991625.1 | *Enterococcus* sp | 42884 |
|  |  |  |  |
| **Group 3** |  |  |  |
| BC-611 | AB712291.1 | *E. faecalis* | 53996 |
| EF-P10 | KY472224.1 | *E. faecalis* | 57408 |
| EF-P29 | KY303907.1 | *E. faecalis* | 58984 |
| Entf1 | MK800154.1 | *E. faecalis* | 58938 |
| IME-EF1 | KF192053.1 | *E. faecalis* | 57081 |
| SAP6 | JF731128.1 | *E. faecalis* | 58619 |
| vB_EfaS_EF1c55 | MN103542.1 | *E. faecalis* | 55876 |
| vB_EfaS_Ef2.2 | MK721189.1 | *E. faecalis* | 58400 |
| vB_EfaS_Ef7.1 | MK721194.1 | *E. faecalis* | 58018 |
| vB_EfaS_HEf13 | MH618488.1 | *E. faecalis* | 57811 |
| vB_EfaS_IME198 | KT932699.1 | *E. faecalis* | 58000 |
| vB_EfaS_PHB08 | MK570225.1 | *E. faecalis* | 55244 |
| VD13 | KJ127303.1 | *E. faecalis* | 55726 |
| EfsWh-1 | MH791415.1 | *E. faecalis* | 58036 |
| vB_EfaS_TV16 | MN939408.1 | *E. faecalis* | 58127 |
| EFC1 | MW677132.1 | *E. faecalis* | 56099 |
|  |  |  |  |
| **Group 4** |  |  |  |
| 9181 | MT939240.1 | *E. faecium* | 71854 |
| 9183 | MT939241.1 | *E. faecium* | 86301 |
| nattely | MT119360.1 | *E. faecalis* | 85669 |
| vB_EfaS_140 | CAJCJZ010000002.1 | *Enterococcus* sp. | 85454 |
| VFW | LT546029.1 | *Enterococcus* sp. | 85865 |
| VPE25 | LT546030.1 | *Enterococcus* sp. | 86524 |
| vipetofem | MT119361.1 | *E. faecalis* | 85371 |
|  |  |  |  |
| **Group 5** |  |  |  |
| 156 | LR031359.1 | *Enterococcus* sp. | 141133 |
| 163 | CAJDKA010000002.1 | *Enterococcus* sp. | 150836 |
| ECP3 | KJ801817.1 | *E. faecalis* | 145518 |
| EF1 | MF001358.1 | *E. faecalis* | 141996 |
| EF5 | MF001361.1 | *E. faecalis* | 141996 |
| EFDG1 | KP339049.1 | *E. faecalis* | 147589 |
| EFLK1 | KR049063.1 | *E. faecalis* | 130952 |
| EFP01 | KY549443.1 | *E. faecium* | 155053 |
| EfV12-phi1 | MH880817.1 | *E. faecium* | 152770 |
| iF6 | MT909815.1 | *E. faecium* | 156592 |
| PBEF129 | MN854830.2 | *E. faecalis* | 141520 |
| PEf771 | MN241318.1 | *E. faecalis* | 151052 |
| phiEF17H | AP018714.1 | *Enterococcus* sp. | 143638 |
| phiEF24C | AP009390.1 | *E. faecalis* | 142072 |
| phiEF24C-P2 | AB609718.1 | *E. faecalis* | 142072 |
| phiM1EF22 | AP018715.1 | *Enterococcus* sp. | 143046 |
| vB_EfaH_149 | CAJDJZ010000002.1 | *Enterococcus* sp. | 142215 |
| vB_EfaH_EF1TV | MK268686.1 | *E. faecalis* | 143507 |
| vB_EfaM_A2 | MT856905.1 | *E. faecalis* | 149431 |
| vB_EfaM_Ef2.1 | MK693030.1 | *E. faecalis* | 140938 |
| vB_EfaM_Ef2.3 | MK721192.1 | *E. faecalis* | 147289 |
| vB_OCPT_Ben | MN027503.1 | *E. faecalis* | 151985 |
| EfsSzw-1 | MH791397.1 | *E. faecalis* | 150272 |
| EFGrKN | MW004544.1 | *E. faecalis* | 147532 |
| EFGrNG | MW004545.1 | *E. faecalis* | 145199 |
| phi EF7H | LC596377.1 | *E. faecalis* | 143399 |
| Phi EF14H | LC596378.1 | *E. faecalis* | 143280 |
| phi EF19G | LC596379.1 | *E. faecalis* | 143400 |
| 113 | MZ147816.1 | *E. faecalis* | 155715 |
| Porthos | LR990835.1 | *E. faecalis* | 153193 |
| vB_EfaM_LG1 | MZ420150.1 | *E. faecalis* | 150025 |
